# Music in the Operating Room: Impact on Team Communication, Focus, and Monitoring Patient Safety

**DOI:** 10.7759/cureus.116131

**Published:** 2026-09-12

**Authors:** Lillian N van Biljon, Zander N Miller, Carsen Steele, Alleana Corpin, Elizabeth R Merwin, Joseph Keebler, Gary Sutkin

**Affiliations:** 1 Department of Surgery, University of Missouri-Kansas City School of Medicine, Kansas City, USA; 2 Department of Human Factors and Behavioral Neurobiology, Embry-Riddle Aeronautical University, Daytona Beach, USA; 3 Department of Obstetrics and Gynecology, University of Missouri-Kansas City School of Medicine, Kansas City, USA

**Keywords:** communication, interprofessional teamwork, music, operating rooms, patient safety

## Abstract

Background

Music is commonly played in the operating room (OR), yet excessive noise is a known contributor to miscommunication and surgical errors. This study examined how music choice and surgical phase influence surgical team members' perceptions of concentration, communication, and patient safety among OR staff.

Methods

A survey was administered via Research Electronic Data Capture (REDCap) in person, by email, and over Zoom to surgeons, nursing staff, and anesthesia providers. Questions addressed music preferences, who selects the music, and perceived effects during routine versus complex phases of surgery. Responses were analyzed by age and role using chi-squared tests.

Results

Response rate was 63.6%. Among 138 respondents (61 surgeons, 40 nursing staff, 37 anesthesia providers), 34.8% (n=48) preferred music at all times, and 10.9% (n=15) preferred none. Music preferences spanned 44 genres, five of which were commonly perceived as disruptive. Although 70.3% (n=97) reported mood benefits, 33% (n=37) reported impaired communication, 8% (n=11) impaired concentration, and 11.6% (n=16) impaired patient safety monitoring. Only 9.4% (n=12) of respondents selected the music; 75% (n=9) of these were attending surgeons (p<0.001), and most did not solicit team preferences. Two-thirds (66.2%; n=49) reported that music appropriateness depended on surgical phase, particularly during high-focus tasks such as counts.

Conclusion

While music is generally favored in the OR, it is centrally controlled and may negatively affect critical aspects of teamwork and safety. Greater awareness of surgical phase and shared decision-making around music selection may improve communication, team dynamics, and patient safety.

## Introduction

Music is commonly played in the operating room (OR), with 60-90% of team members supporting its regular use [1,2]. Reported benefits include improved concentration, teamwork, and perceived calming effects. In a survey of physicians and nurses, approximately two-thirds of respondents believed music had a favorable effect in the OR, and 77% reported that it made them feel calmer and more efficient [2]. Similarly, a systematic review found generally favorable attitudes toward OR music and perceived improvements in individual and team performance [3]. However, concerns remain that music may impair communication [4], reduce vigilance [5], and contribute to the already loud OR environment, a known factor in miscommunication and surgical error [6].

Communication demands vary by surgical phase (e.g., intubation, critical steps, closure), and music preferences may shift accordingly. Prior surveys have examined overall attitudes toward OR music, differences among professional roles, and preferences under selected operative circumstances [1-4,7,8]. However, these studies have not comprehensively evaluated how preferences vary across distinct phases of surgery while also examining the relationship between professional hierarchy, control over music selection, and the expression of team members' preferences. This distinction may be particularly important in the OR, where clear power differentials can facilitate order and efficiency but may also suppress input from lower-status team members [9-11]. Although prior studies suggest that surgeons commonly influence whether and what music is played [1,7], the relationship between professional role, music selection, and team members' ability to voice their musical preferences remains incompletely understood.

We designed the survey to address gaps in the existing literature regarding differences by professional role, preferences across surgical phases, and the effects of music on OR communication. Understanding how preferences differ across roles and how hierarchy shapes music choice may reveal insights into OR culture and psychological safety. We surveyed resident and attending surgeons, anesthesia providers, and nursing staff to examine the relationship between OR hierarchy and music choice, how preferences vary by surgical phase, and how music is perceived to influence communication, concentration, and patient safety.

We hypothesized that most OR team members would prefer music during at least some portions of a surgical procedure and that preferences regarding music type and timing would vary among team members based on multiple contextual factors. We also examined whether professional role and OR hierarchy were associated with differences in music preferences and music selection.

## Materials and methods

Survey development and distribution

The University of Missouri-Kansas City Institutional Review Board approved the study protocol (approval number: 2102629). The survey was developed by an interdisciplinary team that included a medical student, a teaching surgeon, and Human Factors experts. It was constructed based on previous surveys [1-3,5], adding questions from a Human Factors perspective, including portions of surgery, hierarchy/authority, and team communication. Questions included who chooses the music, preferences for music genre and tempo, whether they mind when others choose for the room, and if they ask others their preferences. Other questions included whether music affected concentration, communication, and monitoring patient safety during different portions of routine and complex surgeries and during the critical moment, defined as the phase of the surgery that either most determines the outcome or is at greatest risk for patient injury. 

The final survey (Appendices) was composed of seven demographic questions followed by 25 questions using a combination of multiple-choice with five-point Likert scales and free-text entry. The survey was administered using Research Electronic Data Capture (REDCap) to attending surgeons, anesthesiologists, residents, surgical technicians, circulating nurses, certified registered nurse anesthetists (CRNA), and student registered nurse anesthetists (SRNA). Prior to administration, the survey instrument underwent pilot testing and content validation by a multidisciplinary group consisting of three research assistants, one physician, one medical student, and two certified registered nurse anesthetists, who assessed the survey items for clarity, relevance, and coverage of the intended topics. Primary distribution of the survey was completed in person at department and staff meetings. We also employed snowballing via email. Demographics collected included age, years of experience, department, and role. No financial incentive was offered to complete the survey. 

Quantitative data analysis

Descriptive statistics were reported as frequencies and percentages for categorical variables, means and standard deviations for continuous demographic variables, and frequencies and percentages for Likert-scale items. Analyses were performed using IBM SPSS Statistics for Windows, Version 29.0 (IBM Corp., Armonk, New York, United States). Outcomes were analyzed by age, which correlated with years of experience (Pearson coefficient: 0.799; p<0.001), and role using chi-squared tests. Responses were further analyzed by role. Descriptive data collected by quintiles were analyzed using the midpoint of each interval.

Qualitative data analysis

Prior to survey development, semi-structured interviews were conducted with four OR team members (two surgeons, one circulating nurse, and one CRNA) to identify relevant themes and inform the content of the survey questions. Following survey administration, the free-text survey responses underwent thematic analysis [12]. The first author performed the initial coding and presented the resulting codes with supporting examples to the full research team. Through discussion, the research team compared and refined the codes and reached consensus on the final themes, which were used to contextualize the quantitative survey findings.

## Results

Of the 217 OR team members who received the survey by email or attended a departmental meeting at which it was distributed, 138 completed the survey, resulting in an overall response rate of 63.6%. Respondents included 61 surgeons, 40 nursing staff, and 37 anesthesia providers. The surgical attendings and residents were specializing in General Surgery (21), Orthopedics (12), Obstetrics and Gynecology (12), Oral and Maxillofacial Surgery (OMFS) (8), Urology (5), Otorhinolaryngology (ENT) (2), and Plastic Surgery (1). Most respondents (61.6%; n=85) were female, and the estimated mean age was 38.7 years (SD 11.4). Nearly half (47.4%; n=65) were 35 or younger. Respondents aged 51 or older comprised 16.7% (n=23) of the sample, including 33.3% (n=7) of surgical attendings, none of the surgical residents, 13.5% (n=5) of anesthesia providers, and 27.5% (n=11) of nursing staff and other. A total of 14.6% (n=20) of respondents reported 20 or more years of OR experience (Table 1). 

**Table 1:** Demographics: roles, gender, age, and experience *N (%). Three respondents did not report gender. ​​**One respondent was excluded from the age calculation due to an erroneous entry​​​. ​​***Estimated means (standard deviations). ****Other includes educational professionals and research personnel.

Role	​​​Age (years)***	​​​Gender* (female)	​​​Years of experience***
Surgical attending	47.5 (11.3)	7 (33.3)	16.5 (5.9)
Surgical resident	30.1 (3.7)	20 (50)	3.5 (2.8)
Anesthesiology attending	43.5 (7.8)	3 (23.1)	13.4 (5.6)
Anesthesiology resident	34.4 (4.4)	6 (66.7)	4.7 (4.2)
Certified registered nurse anesthetist	47.5 (13.5)	10 (100)	15.5 (7.1)
Student registered nurse anesthetist​	42.5 (8.7)	4 (57.1)	2.5 (0)
Circulating nurse	38.8 (13.5)	22 (84.6)	11.5 (6.9)
Surgical technician	51.3 (10.3)	10 (90.9)	10.2 (4.5)
Other****	31.9 (4.6)	3 (100)	12.5 (7.1)
Total**	38.7 (11.4)	85 (61.6)	9.4 (7.3)

Musical preferences were highly individualized: 91 (65.9%) respondents preferred 44 separate genres (Figure 1). Pop music was the most preferred genre, while the genres of rap, scream-o, heavy metal, club, and country music were reported by some as unfavorable. Most participants (70.3%; n=97) said music improved their mood, and 29.5% (n=41) felt it enhanced efficiency. Regarding tempo, 8.5% (n=9) preferred fast, 42.5% (n=45) medium, and 4.7% (n=5) slow, while 44.3% (n=47) had no preference. A majority (67.4%; n=93) of participants reported listening to music in most surgeries. Professional role was significantly associated with whether respondents would choose to play music during surgical procedures if they were responsible for the music (χ²(16, N=138)=30.69; p=0.015; Cramér's V=0.33). However, because 66.7% of cells had expected counts below five, including a minimum expected count of 0.33, the assumptions of the chi-squared test were not met, and this exploratory finding should be interpreted cautiously. Concerning duration, 34.8% (n=48) of respondents preferred music to be playing the entire time, including 33.3% (n=7) of surgical attendings, 50% (n=20) of surgical residents, 36.4% (n=4) of surgical technicians, and 55.6% (n=5) of anesthesia residents. Conversely, 10.9% (n=15) of respondents preferred no music to be played for the entire surgery, including 28.6% (n=6) of surgical attendings, 18.2% (n=2) of anesthesiology attendings, 15.4% (n=4) of circulating nurses, and 27.3% (n=3) of surgical technicians. 

**Figure 1:**
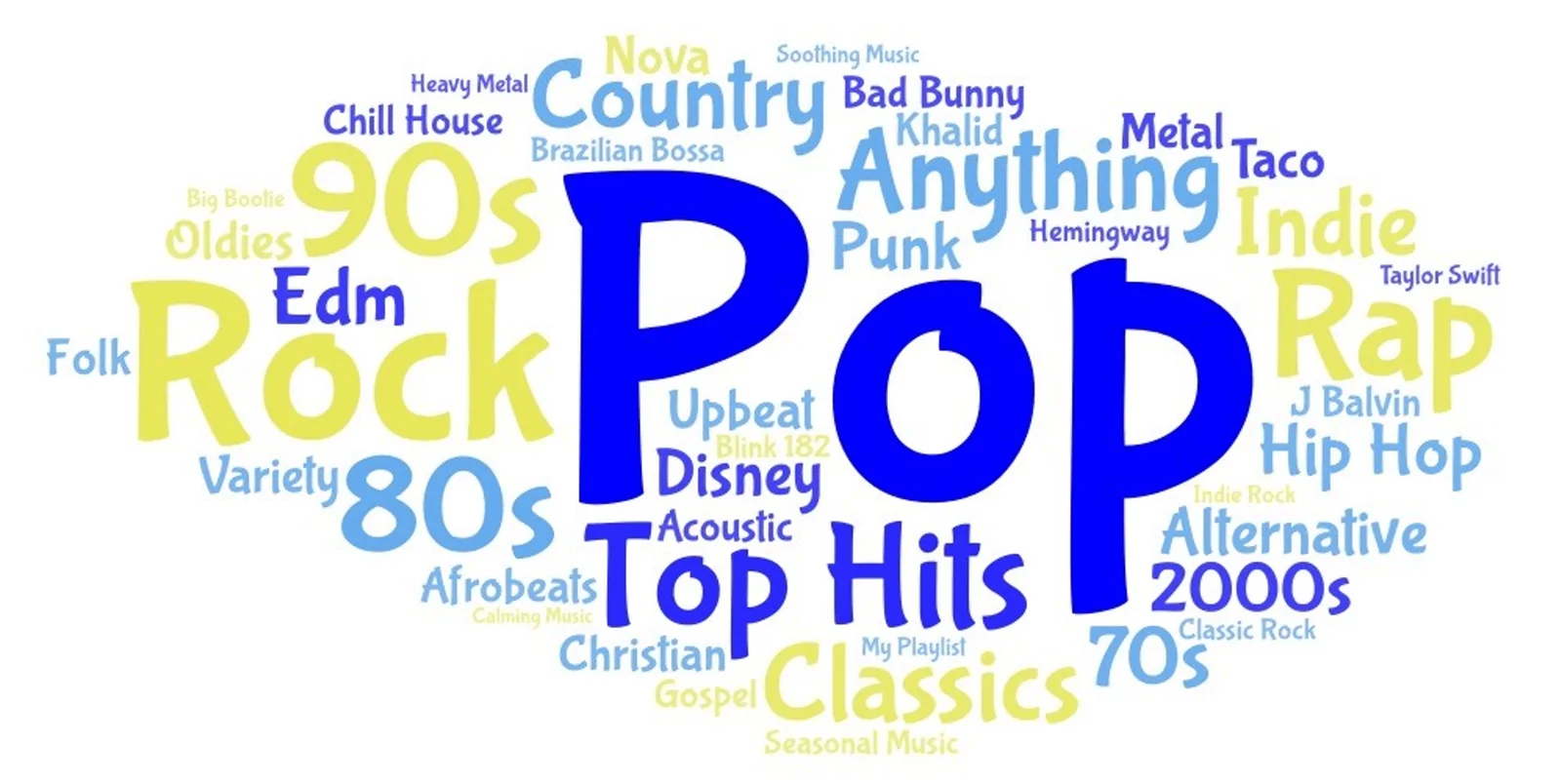
Word cloud representing staff preferred music genres Word size corresponds to the frequency with which each term appeared. The figure was created by the authors using the manual version of WordArt.com (https://wordart.com).

When asked about their preference for music in the OR, 54.3% (n=75) responded that it "depends", and of those, 66.2% (n=49) reported it depends on the portion of the surgery (Table 2): 70% (n=3) of all surgeons (including attendings and residents), 92.8% (n=13) of nursing staff, and 100% (n=15) of anesthesia providers agreed on wanting no music during the critical moment of the surgery. By contrast, 96% (n=39) of participants who wanted at least some music wanted it played during routine portions of the surgery. Regarding the count, 64.3% (n=9) of the nursing staff and 35% (n=7) of surgeons preferred no music. Anesthesia providers preferred no music during intubation (100%; n=15) and extubation (93.3%; n=14). During timeout, 100% (n=49) of all respondents wanted music turned off, and 48.7% (n=58) wanted it off for the duration of complex surgeries. 

**Table 2:** Operating room team members who preferred "no music" during specific phases of surgery All values are presented as n (%). *Other includes educational professionals and research personnel.

Role	Setup	Intubation	Critical moment	Routine portions	The count	Extubation
Surgical attending	2 (28.6)	5 (71.4)	3 (42.9)	0 (0)	1 (14.3)	3 (42.9)
Surgical resident	4 (30.8)	13 (100)	11 (84.6)	1 (7.7)	6 (46.2)	8 (61.5)
Anesthesia attending	1 (16.7)	6 (100)	6 (100)	0 (0)	0 (0)	5 (83.3)
Anesthesia resident	0 (0)	4 (100)	4 (100)	1 (25)	2 (50)	4 (100)
Student registered nurse anesthetist	1 (100)	1 (100)	1 (100)	0 (0)	0 (0)	1 (100)
Certified registered nurse anesthetist	1 (25)	4 (100)	4 (100)	0 (0)	2 (50)	4 (100)
Circulating nurse	1 (11.1)	8 (88.9)	8 (88.9)	0 (0)	6 (66.7)	8 (88.9)
Surgical technician	0 (0)	2 (100)	2 (100)	0 (0)	1 (50)	1 (50)
Other*	0 (0)	2 (66.7)	3 (100)	0 (0)	2 (66.7)	2 (66.7)
Total	10 (20.4)	45 (91.8)	42 (85.7)	2 (4.1)	20 (40.8)	36 (73.5)

Regarding performance, some participants reported that music consistently worsened their ability to communicate (33%; n=37), concentrate (8%; n=11), and monitor patient safety (11.6%; n=16), especially with higher music volume. Table 3 reports these data by role. However, a majority of respondents indicated that music does not impact key aspects of their work: 67% (n=75) reported improvement or no effect on communication, 55.1% (n=76) stated the same for concentration, and 62.3% (n=86) said the same for monitoring patient safety. 

**Table 3:** Operating room team members reporting that music worsens communication, concentration, and monitoring patient safety All values are listed as % (n) of respondents to that question.

Role	Worsens communication	Worsens concentration	Interferes with monitoring patient safety
Surgical attending	46.7 (7)	9.5 (2)	23.8 (5)
Surgical resident	25 (9)	0 (0)	10 (4)
Anesthesia attending	18.2 (2)	18.2 (2)	0 (0)
Anesthesia resident	33.3 (3)	11.1 (1)	11.1 (1)
Student registered nurse anesthetist	14.3 (1)	14.3 (1)	0 (0)
Certified registered nurse anesthetist	40 (4)	10 (1)	20 (2)
Circulating nurse	43.8 (7)	7.7 (2)	3.8 (1)
Surgical technician	42.9 (3)	9.1 (1)	18.2 (2)
All respondents	33 (37)	8 (11)	11.6 (16)

Only 9.4% (n=12) of respondents reported that they were the ones who usually choose the music, while 90.6% (n=115) reported that someone else chooses the music. Staff role was significantly associated with who chooses the music (χ²(8, n=127)=48.44; p<0.001; Cramér's V=0.618). About 75% (n=9) of those in our sample who chose the music were attending surgeons, and 58.3% (n=7) were over the age of 40. No anesthesia providers or surgical technicians reported themselves as choosing. Around 21% (n=29) do not like others choosing the music. Among respondents younger than 40, 94.2% (n=81) said others choose the music, and 72.2% (n=65) find those choices favorable (Figure 2). Nearly one in five surgical attendings and residents (19.6%; n=11) identified the circulating nurse as the music selector, though only 9.5% (n=2) of the circulating nurses reported choosing. Most anesthesia providers and nursing staff (90.3%; n=34) said that surgeons were the ones who selected the music. Only 33.3% (n=7) of attending surgeons who regularly choose the music routinely ask others about their preference.

**Figure 2:**
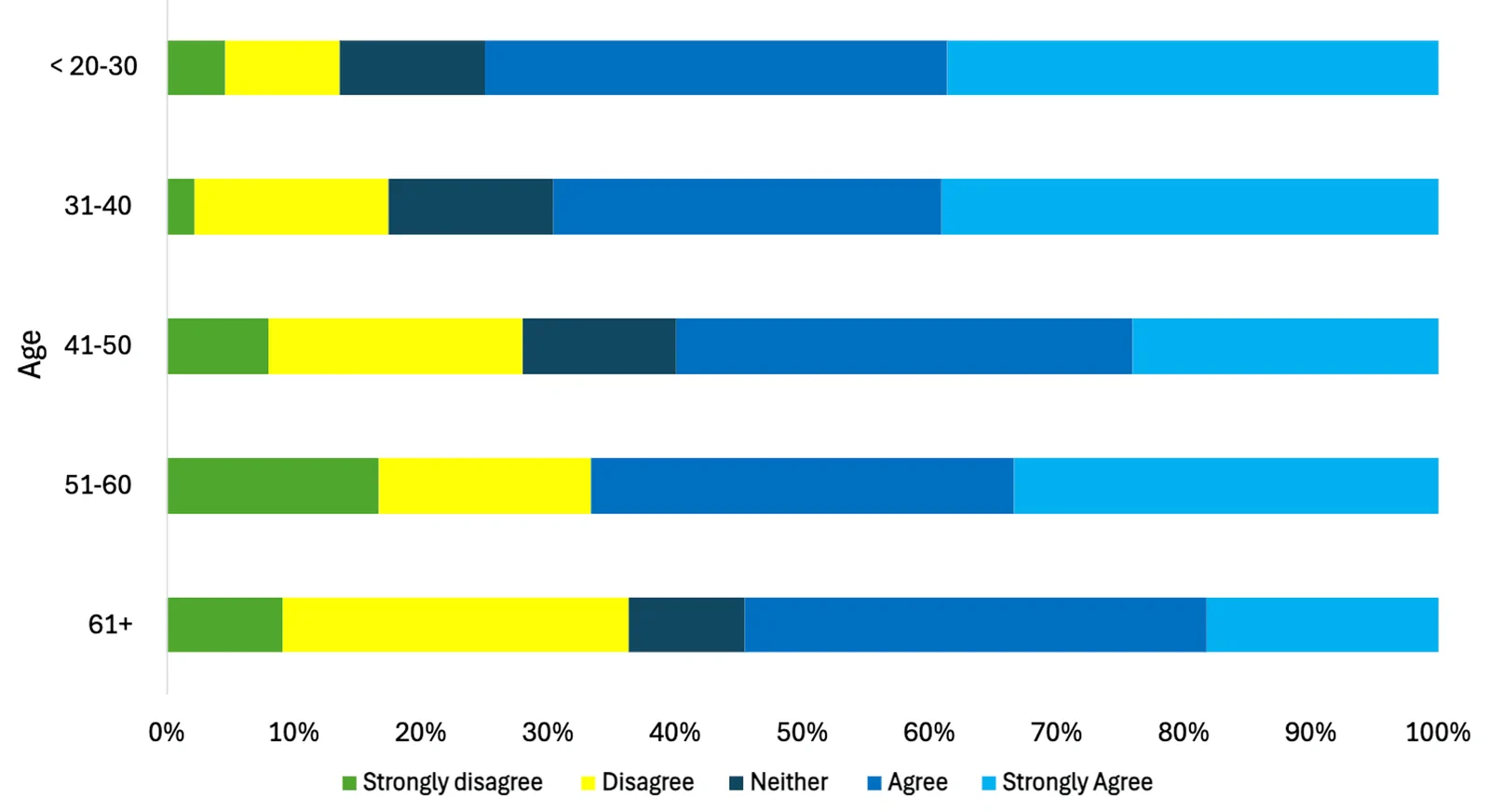
Acceptance of others' music choices Bar chart dictating how agreeable the age groups are to the statement "It doesn't matter to me what kind of music is played; whatever music others choose is fine with me".

Qualitative results 

Interviews with OR staff members revealed several themes regarding music in the OR. Many expressed a desire to turn off music during complex or high-stress portions of procedures, particularly when precise verbal communication was necessary. One surgeon noted, "Normally we would be listening to music, but today we are not going to because we … need to talk a lot during this case and I want to make sure everyone can hear everyone clearly". Interviewees emphasized that music could interfere with verbal communication, especially during handovers or communicating across the room. Tension also arose around some attending surgeons' musical choice. A CRNA shared, "Listening to that type of music at a loud volume becomes a distraction for me mentally so that takes away my focus from the patient". Staff expressed hesitation to request the music be turned down or off, even when it was distracting or raised safety concerns. As one circulating nurse reflected, "I do understand that the surgeon likes the music on because it helps drown out the monotony". Finally, louder music was sometimes described as interfering with the ability to hear alarms or monitors; a CRNA compared it to "driving a car with earplugs in".

## Discussion

In our study, music was commonly played during surgery and preferred by most participants, who reported improved mood and efficiency. The attending surgeon typically decided whether and what music was played, and these decisions were generally accepted by the team. However, a notable minority preferred no music, some only during specific portions, such as residents during critical moments or circulating nurses during the count, and others throughout the entire case. To our knowledge, these phase-specific music preferences have not been previously reported.

Communication and patient safety 

Aversion to music in the OR often stems from concerns that it interferes with critical tasks such as communication and patient monitoring. The OR is already noisy [6], and music adds to this burden, threatening team coordination and communication, reducing auditory perception [13,14], and increasing repeated requests [4]. One survey found that 25% of anesthesia providers felt music disrupted communication or safety [5]. Although only 8% of our participants reported impaired concentration, even a small minority matters during high-focus moments like intubation, the critical moment, the count, or extubation. Open discussion about music use and volume may strengthen communication, teamwork, and safety in the OR.

Hierarchy and psychological safety 

In one survey, 80% of anesthesia and nursing staff believed music choice should be shared, while half of surgeons felt it was their decision alone [7]. We observed a similar pattern: attending surgeons rarely solicited input from others. This dynamic affects teamwork and psychological safety. Although our study did not evaluate whether participants raised patient safety concerns, we hypothesize that discomfort with voicing opinions on low-stakes issues such as music could reflect broader hierarchical barriers to speaking up in high-stakes, noisy OR situations [9-11]. Moreover, when music interferes with communication, concentration, or safety monitoring, requesting that it be lowered or turned off may itself represent a direct patient-safety action rather than merely an expression of personal preference. Explicitly empowering all OR team members to request modification of music may help overcome hierarchical barriers and promote timely communication.

Music and productivity

Music in the workplace is common across industries such as hospitality, retail, manufacturing, and office settings [15-17]. It can influence mood, satisfaction, and performance, though not always positively [17]. Task type and music style play key roles: for repetitive, low-cognitive tasks, such as exercise training, music can enhance motivation and performance [18], while for complex tasks, certain genres may impair focus [19]. Just as drivers lower music volume in heavy traffic, OR staff may benefit from silence during demanding surgical phases.

Music type also affects performance. Pleasant, stimulating music improves reaction time and engagement [20], whereas loud music can be distracting and increase repeated communication [19]. Self-selected music enhances efficiency in simulated surgical tasks compared to typical noisy OR conditions [21]. Our participants' genre and tempo preferences varied widely, mirroring general population patterns [16]. These findings suggest that collaborative music selection may enhance overall OR efficiency and team harmony.

Limitations

There are several limitations to this study. Although the response rate was 63.6% (n=138), selection bias remains possible because nonrespondents may have held different views about music. This cross-sectional study was conducted at a single teaching institution, which may limit the generalizability of the findings to other institutions and ORs without learners and prevents causal inference. Furthermore, the study relied on participants' self-reported perceptions rather than objective measures of communication quality, cognitive performance, OR efficiency, patient safety, or clinical outcomes. Psychological safety and speaking-up behaviors were not directly assessed using validated measures; therefore, interpretations regarding these concepts should be considered hypotheses for future investigation. The REDCap branching logic excluded participants who preferred music throughout the entire surgery or no music at all from certain questions, including those concerning preferences during specific operative phases. Future surveys should assess whether these participants have exceptions to their overall preferences during particular phases. Finally, the qualitative findings were based on only four exploratory interviews intended to inform survey development rather than provide a comprehensive qualitative analysis. Thematic saturation was not assessed, and the small interview sample may not represent the full range of perspectives among OR team members.

## Conclusions

While music in the OR is generally well-received, some team members report that it hinders their work, particularly during high-focus phases or when volume is excessive. Although many accept the attending surgeon selecting the music, others prefer greater input. These findings support greater awareness of operative phase, volume, and differing team member preferences when using music in the OR. The team's music choices may offer insight into psychological safety and the culture of speaking up for patient safety. Future research should examine how those who choose music respond when asked to turn it off and how team dynamics influence the willingness of others to speak up. 

## Appendices

Survey

1. To help us match your responses in this survey with a follow-up survey, we kindly ask for your first and last name. Please be assured that your name will be used solely for this purpose and will remain confidential. Your identity will not be disclosed, and all your responses will be anonymized in any reports or publications. Thank you for your participation.

     a. Name: ______________________

2. Your age (in years): 

     a. Less than 20

     b. 20-25

     c. 25-30

     d. 30-35

     e. 35-40

     f. 45-50

     g. 55-60

     h. 60-65

     i. More than 65

3. What is your gender identity? 

     a. Female 

     b. Male 

     c. Transgender

     d. Non-binary/non-conforming

     e. Other: ______________ (free text)

     f. Prefer not to respond

4. What is your ethnic background? Please check all that apply.

     a. White/Caucasian

     b. Asian: Eastern

     c. Asian: Indian

     d. Hispanic

     e. Black

     f. Native American

     g. Prefer not to answer

     h. Other: _______________ (free text)

5. Position (Please choose the role you fulfill most often)

     a. Surgical attending

     b. Surgical resident

     c. Anesthesiology attending

     d. Student registered nurse anesthetist

     e. Certified registered nurse anesthetist

     f. Circulating nurse 

     g. Surgical technician 

     h. Medical student

     i. Other: _______ (free text)

6. If you are an attending or resident surgeon, please identify the subspecialty you most identify with: 

     a. General Surgery 

     b. Plastic Surgery 

     c. Orthopedics

     d. ENT

     e. Ophthalmology

     f. Chest-Heart Surgery

     g. Neurosurgery

     h. Obstetrics/Gynecology

     i. Maxillofacial Surgery

     j. Vascular Surgery 

     k. Urology 

     l. I am not a surgeon 

7. How long have you been working in an OR? 

     a. Less than 5 years 

     b. Between 5 and 10 years

     c. Between 10 and 15 years

     d. Between 15 and 20 years

     e. More than 20 years 

8. Please estimate the percentage of surgical procedures you participate in where music is played for a majority of the surgery:

     a. None

     b. 0-20%

     c. 21-40%

     d. 41-60%

     e. 61-80%

     f. 81-100%

If they answer "a", skip to question 12.

If they answer "b-f", skip to question 9.

9. In your experience at UH, who most commonly chooses the music in the OR? 

     a. I most commonly choose the music 

     b. Someone else commonly chooses the music

If they answer "b", skip to question 11.

If they answer "a", proceed to question 10.

10. Who usually chooses the music?

     a. The attending surgeon 

     b. The resident surgeon

     c. The certified registered nurse anesthetist

     d. The circulating nurse

     e. The surgical technician

     f. The patient  

     g. Other: _____ (free text)

11. When you choose (or if you were able to choose) the music for a surgical case, which music would you choose? 

     a. (Open-ended response here)

12. Do you agree with this sentence?

     "It doesn't matter to me what kind of music is played; whatever music others choose is fine for me".

     a. Strongly agree 

     b. Slightly agree 

     c. Neither agree nor disagree 

     d. Slightly disagree 

     e. Strongly disagree

13. How often, prior to the start of a case, do you ask others in the OR what their music preferences are?

     a. Never

     b. Less than half of the cases

     c. About 50% of the cases

     d. More than half of the cases

     e. Every case

14. If you were in charge of the music in the OR, would you choose it to be played during surgical procedures? 

     a. Yes, all the time, in every surgery

     b. No, I prefer no music to be played during any surgery

     c. I like music sometimes and no music other times. It depends

If #14 is answered "a. Yes", the survey will go to question #26 (see above: type of music).

If #14 is answered "b. No", the survey will go to question #26.

If #14 is answered "c. I like music sometimes and no music other times. It depends", the subject will continue to the next question, #15. 

15.  Does the portion of the surgery impact whether you prefer music to be played? 

     a. Yes, I prefer music during certain portions

     b. No, the portion of the surgery does not impact my music preference

If they answer "b. No" to #15, then they skip #16-25 and move on to #26.

If they answered "a. Yes", they move on to #16-25.

16.  OR setup with patient out of the room

     a. I prefer music

     b. I prefer some music

     c. I prefer no music

     d. I have no preference

17.  Patient enters the room until surgery starts (excluding intubation and timeout) 

     a. I prefer music

     b. I prefer some music

     c. I prefer no music

     d. I have no preference

18.  Intubation

     a. I prefer music

     b. I prefer some music

     c. I prefer no music

     d. I have no preference

19.  Timeout

     a. I prefer music

     b. I prefer some music

     c. I prefer no music

     d. I have no preference

20.  The critical moment when the outcome and patient safety most depend on focused tasks, monitoring the safety of the patient, and coordinated communication 

     a. I prefer music

     b. I prefer some music

     c. I prefer no music

     d. I have no preference

21.  More routine portions of the surgery (i.e., not the critical moment)

     a. I prefer music 

     b. I prefer some music

     c. I prefer no music

     d. I have no preference

22.  The count (instruments/laps) 

     a. I prefer music

     b. I prefer some music

     c. I prefer no music

     d. I have no preference

23.  The end of the surgery (i.e., "closing") 

     a. I prefer music

     b. I prefer some music

     c. I prefer no music

     d. I have no preference

24.  Extubation

     a. I prefer music

     b. I prefer some music

     c. I prefer no music

     d. I have no preference

25.  Is there any other point in the surgery that affects your music preference?

     a. (Open-ended response here)

26.  Does your familiarity with the procedure impact whether you prefer music to be played? 

     a. If I am more familiar, I would prefer to have the music on

     b. If I am less familiar, I would prefer to have the music off

     c. If I am more familiar, I would prefer to have the music off

     d. If I am less familiar, I would prefer to have the music on

     e. My familiarity does not affect my feelings about music being on or off

27.  Do you prefer no music when you are unfamiliar with the procedure? 

     a. If I am more familiar, I would prefer to have the music on

     b. If I am less familiar, I would prefer to have the music off

     c. If I am more familiar, I would prefer to have the music off

     d. If I am less familiar, I would prefer to have the music on

     e. My familiarity does not affect my feelings about music being on or off

28.  Do you prefer no music when you anticipate the procedure to be more complex or unpredictable? 

     a. If the procedure is anticipated to be more complex/unpredictable, I would prefer to have the music on

     b. If the procedure is anticipated to be more complex/unpredictable, I would prefer to have the music off

     c. The procedure's complexity or unpredictability does not affect my feelings about music being on or off

29.  Does familiarity with your coworkers in the room impact whether you prefer music to be played?

     a. If I am more familiar, I would prefer to have the music on

     b. If I am less familiar, I would prefer to have the music off

     c. If I am more familiar, I would prefer to have the music off

     d. If I am less familiar, I would prefer to have the music on

     e. My familiarity does not affect my feelings about music being on or off

30.  When music is played, do you prefer the volume to be:

     a. Loud

     b. Medium

     c. Quiet

     d. No preference

31.  When music is played, do you prefer the tempo to be:

     a. Fast

     b. Medium

     c. Slow

     d. No preference

32.  How does your mood influence if you want music played? (Check all that apply)

     a. If I am in a good mood, I usually want music to be played

     b. If I am in a bad mood, I usually don't want music to be played

     c. If I am in a good mood, I usually don't want music to be played

     d. If I am in a bad mood, I usually want music to be played

     e. No, my mood does not influence if I want music to be played

33.  How does music in the OR affect your ability to communicate to others and for them to communicate with you? 

     a. Music improves my ability to communicate with others

     b. Music worsens my ability to communicate with others

     c. Music has no effect on my ability to communicate with others

34.  How does music in the OR affect your ability to concentrate on tasks? 

     a. Music improves my ability to concentrate

     b. Music worsens my ability to concentrate

     c. Music has no effect on my ability to concentrate

     d. It depends (directed to 34a) 

34a. Please describe how music in the OR affects your ability to concentrate on tasks 

(Text Box) 

35.  How does music in the OR affect your ability to monitor patient safety? 

     a. Music improves my ability to monitor patient safety

     b. Music worsens my ability to monitor patient safety

     c. Music has no effect on my ability to monitor patient safety

     d. It depends (directed to 35a) 

35a. Please describe how music in the OR might affect your ability to monitor patient safety 

(Text Box) 

36.  How does music in the OR affect your mood? 

     a. Music improves my mood

     b. Music worsens my mood

     c. Music has no effect on my mood

37.  How does music in the OR affect your efficiency? 

     a. Music improves my efficiency

     b. Music worsens my efficiency

     c. Music has no effect on my efficiency
